# Hypertrophic Scar Formation on Application of Terpenoid Fraction of Tuberous Root of *Mirabilis jalapa* L. on Excision Wound Model in Wistar Albino Rats

**DOI:** 10.1155/2014/583730

**Published:** 2014-11-13

**Authors:** Jyotchna Gogoi, Khonamai Sewa Nakhuru, Pronobesh Chattopadhayay, Ashok Kumar Rai, Vijay Veer

**Affiliations:** ^1^Defence Research Laboratory, Post Bag No. 2, Tezpur, Assam 784001, India; ^2^Department of Life Sciences, Dibrugarh University, Dibrugarh, Assam 786004, India

## Abstract

The study was designed to evaluate the effects of hydromethanolic extract of tuberous root of *M. jalapa* and its terpenoid and flavonoid fractions on cutaneous wound healing in Wistar Albino rats. The hydromethanolic extract was subfractionated by sequential extraction in solvents (moderately nonpolar to polar). The extract and its (terpenoid and flavonoid) fractions were used for cutaneous wound healing studies by using excision wound model on rat. Their effects on wound contraction rate, biochemical and histological changes, and expression of growth factors such as collagen 3A, basic fibroblast growth factor, and vascular endothelial growth factor were investigated. The results indicated that flavonoid treated group showed significant decrease (*P* < 0.05) in antioxidant enzyme level as compared to control in wound healing process, whereas terpenoid fraction showed significant increase (*P* < 0.05) in expression of growth factor levels but regeneration and remodeling stages were delayed due to formation of thicker ulcus layer and also there were no hair follicle-like blood capillaries formation which ultimately may lead to formation of hypertrophic scar of wound. Therefore, from this study, it can be concluded that terpenoid fraction prolongs proliferation phase and hence may have tendency to convert the wound into hypertrophic wound.

## 1. Introduction

*Mirabilis jalapa* L. (Nyctaginaceae) is widely used as a traditional folk remedy for treating various ailments including acute arthritis and inflammation. Chemical investigation of* M. jalapa* has shown the presence of fatty acids [1], terpenoids, steroids [2], and D-glucan [3]. Major sterol such as *β*-sitosterol, daucosterol, and *β*-sitosterol acetate from all the parts of this herb has been reported. Phenolic compound 2′-O-methylabronisoflavone with activity against* Candida albicans* was isolated from tissue cultured* M. jalapa* [4]. Four new rotenoids mirabilijalones (A, B, C, and D) and 1,2,3,4-tetrahydro-1-methylisoquinoline-7,8-diol with anti-HIV activity were isolated from* M. jalapa* root [5]. Tuberous root of* M. jalapa* has been attributed to* in vivo* antimicrobial activity [6].

Traditionally, few tribes of Senapati district of Manipur (India) use* M. jalapa* roots for washing cuts and curing injury [7].* M. jalapa* tuberous root extract showed cutaneous wound healing potential on both excision and dead space wound models in Wistar albino rat [6]. Phytochemicals such as terpenoids, phenolics, alkaloids, and flavonoids were detected in the crude extract of* M. jalapa*. Upon fractionation, terpenoid and flavonoid fractions showed better antioxidant and antimicrobial activities as compared to crude extract [8]. Thus, in the present study, comparative wound healing activity of terpenoid and flavonoid fractions and crude extract of tuberous root of* M. jalapa* was evaluated on excision wound model in Wistar albino rat.

## 2. Materials and Methods

### 2.1. Chemicals and Reagents

Ascorbic acid, 2,2′-azino-bis(3-ethylbenzothiazoline-6-sulfonic acid) (ABTS), acrylamide, ammonium persulphate (APS), Tris-buffered saline, and glycine were purchased from Sigma Aldrich. Polyvinylidene fluoride (PVDF) membrane was from Bio-Rad, CA, USA. Antibodies were from Santa Cruz. Western blotting development kit was purchased from Ameresco. Silverex ointment was from Rexcin Pharmaceuticals Pvt. Ltd., India. Other chemicals were analytical grade and purchased from Merck and HiMedia, India.

### 2.2. Plant Material

*M. jalapa* (pink flowers) were collected from locally grown flower gardens of Tezpur (91° 48′E and 26° 38′N), Assam. Voucher specimen was authenticated by the Botanical Survey of India (BSI), Shillong (number BSI/ESC/2011/Plant identification/93).

### 2.3. Extraction and Fractionation

Tuberous root of* M. jalapa* was washed with tap water, shade-dried for two weeks, and powdered in a grinder. 100 g of powdered sample was extracted with 1000 mL of 80% methanol for 48 h. Repeated percolation was done. Extracts were pooled and filtered by using Whatman No. 1 filter paper. Filtrate was concentrated under reduced pressure in rotary vacuum evaporator (RV10 Control, IKA, Germany). Concentrated hydromethanolic extract of* M. jalapa* (MJE) was air-dried to a constant weight at room temperature and stored in −20°C refrigerator for further studies.

Fractionation of the MJE was done based on polarity of the solvents. Chloroform, ethyl acetate, isoamyl alcohol, methanol, and water were used for fractionation [8]. Four fractions were collected, out of which terpenoid fraction (F1) and flavonoid fraction (F3) showed better antioxidant and antimicrobial activities than those of MJE [8] and thus were selected for the present study. Presence of terpenoids and flavonoids was confirmed by spraying the developed thin layer chromatography plate with KMnO_4_ and vanillin-H_2_SO_4_ sprays for F1 and F3 fractions, respectively. ATR (IR) spectra of MJE, F1, and F3 were recorded on ATR Nicolet IS5 supplied by Thermo Fisher Scientific (Verona Road, Madison, USA) with scanning speed of 1 cm/sec and 32 numbers of scans. Ursolic acid was used as triterpenoid standard for IR spectral analysis.

### 2.4. Wound Healing Activity

#### 2.4.1. Animals

Wistar albino rats (male), weighing 150 ± 10 g, used for the study, were housed in cage individually under controlled conditions of 12 h light/12 h dark cycle and at a temperature of 22 ± 2°C, with free access to rat/mice feed (Lipton, India) and water* ad libitum*. The experimental protocols followed were in accordance with the NIH guidelines and approved by the Institutional Animal Ethical Committee (Reg. number 1127/bc/07/CPCSEA) with Resolution (number 02/Scientist/Agrotech/DRL/2010-11). No toxicity was observed [6].

#### 2.4.2. Excision Wound Model

Excision wound model was used to evaluate the wound healing activity of the extract and fractions. Animals were anaesthetized with ketamine (25 mg/Kg, i.p.) for short duration. The hairs of the rat on the back were depilated by careful shaving. An average wound size of 2.5 cm in diameter (full thickness of skin) was excised on a predetermined area by using toothed forceps and fine scissors [9].

#### 2.4.3. Dose Selection for Comparative Study

Wound contraction rate, epithelialisation period, and malondialdehyde (MDA) level were the parameters used for selection of single dose of F1 and F3 for comparative study of wound healing on excision wound model. 5% ointment formulation of MJE was found to be enhancing wound healing process [6]; thus, 5% ointment formulation was selected for comparative study. For F1, doses ranged from 0.1% to 0.3% and for F3, it ranged from 0.2% to 0.5% were used for selection of single dose. Mentioned doses were calculated based on the amount of F1 and F3 fractions present in 5% of MJE. Each dose was prepared in petroleum jelly base on weight by weight basis. Each treated group comprised of three rats. Based on the changes in the mentioned parameters, dose of 0.2% of F1 and dose of 0.4% of F3 were selected for comparative study of wound healing efficacy on excision wound model.

In the comparative study, rats were divided into five groups with five rats each. Control group was treated topically with petroleum jelly, whereas standard group was treated with silver sulfadiazine (Silverex). Experimental groups were treated topically with 5% (w/w) of MJE, 0.2% (w/w) of F1, and 0.4% (w/w) of F3. Dosing was given topically once a day till the wound was completely healed. The progressive changes in wound area were monitored by a camera (Canon Ixus 105) every four-day interval. Wound area was evaluated by using ImageJ software. Wound contraction was calculated as percentage of the reduction in wounded area.

#### 2.4.4. Biochemical and Histological Evaluation

Granulation tissue was harvested on 8th postwounding day for biochemical and histological evaluation, whereas tissue harvested on 12th postwounding day was used for histological studies. Tissue was divided into five equal parts. One part was made to 10% homogenate in 0.1 M phosphate buffer (pH 7.4) containing 1 mM EDTA and centrifuged at 12000 rpm for 5 min. The collected supernatant was used for enzyme assays and protein estimation. Total protein was determined by Bradford method [10] and bovine serum albumin (BSA) was used as reference standard. Superoxide dismutase (SOD) was determined by Beauchamp and Fridovich [11] method, catalase (CAT) by Aebi [12] method, and peroxidase (POD) by Duarte-Vázquez et al. [13] method. The expression of collagen type III alpha (COL 3A), basic fibroblast growth factor (FGF 2), and vascular endothelial growth factor C (VEGF C) was determined by immunoblotting technique for which 20 *μ*g crude proteins was loaded on to each well of 12.5% SDS polyacrylamide gel and subjected for separation at 25 mA for 2 h. The separated bands were then transferred to polyvinylidene fluoride (PVDF) membrane (Bio-Rad, CA, USA). The nonspecific proteins were blocked by 5% BSA and incubated with primary antibodies (Col 3A, VEGF C, and FGF 2). Alkaline phosphatase conjugated secondary antibodies were used and developed with 5-bromo-4-chloro-3-indolyl phosphate dipotassium/nitrotetrazolium blue chloride (BCIP/NBT) liquid substrate system [14]. The intensity of the blots was analyzed by National Institutes of Health ImageJ software (vl. 38).

*Hydroxylproline Estimation*. One part of the harvested tissue was used for hydroxylproline estimation. The wet weight of the first part was noted and then dried at 60°C for 12 h and the dry weight was recorded. The dried tissue was hydrolysed with 6 N HCl and kept at 110°C for 24 h. The neutralized acid hydrolysate was used for the determination of hydroxyproline according to Neumann and Logan [15].

*Ascorbic Acid Content*. For the estimation of ascorbic acid content, the tissue was homogenized in 6% TCA (1 : 20) and centrifuged at 10000 rpm for 5 min at 4°C. Coloured complex formed by the reaction of ascorbic acid in the supernatant with dinitrophenylhydrazine (DNPH) was read spectrophotometrically at 530 nm [16].

*Lipid Peroxidation*. The granulation tissue was homogenized in 10% TCA (1 : 10) [17] and centrifuged at 12000 rpm for 5 min at 4°C. MDA, a product of lipid peroxidation, was measured by reacting with thiobarbituric acid (TBA) to give a colored substance. This coloured adduct was read at 532 nm. 

*Histology.* Fifth tissue part of 8th and 12th postwounding day tissues was used for histological evaluation. Samples were fixed in 10% buffered formalin, processed and blocked with paraffin, and then sectioned into 5 *μ*m sections by using microtome (Spencer 1010 SMT-006, New Delhi, India) and stained with hematoxylin and eosin (HE) stain [18]. Sections were observed under light microscope (Leica DM 750 attached with Leica Microsystems CH-9453, Switzerland).

### 2.5. Statistical Analysis

Results are presented as mean ± standard deviation (SD); statistical analysis was done using one-way ANOVA followed by Dunnett's multiple comparison test in Graphpad Prism version 5. *P* value ≤ 0.05 was considered significant.

## 3. Results

### 3.1. IR Spectra of MJE and F1 and F3 Fractions

Fractionation yield was about 32.95 ± 10.11 mg of F1 and 58.54 ± 7.69 mg of F3 in 1 g of MJE. KMnO_4_ spray showed the presence of triterpenoids and sterols with yellow coloured spots against purple background in F1 (solvent system : petroleum ether : chloroform : methanol), whereas flavonoids dominant in F3 (solvent system : chloroform : ethyl acetate : formic acid) showed yellow and grey coloured spot on spraying TLC chromatogram with vanillin-H_2_SO_4_ spray. TLC chromatogram of F1 showed separation of two spots prominently, one of which corresponds to *β*-sitosterol. TLC chromatogram of F3 showed separation of two spots.

**Figure 1:**
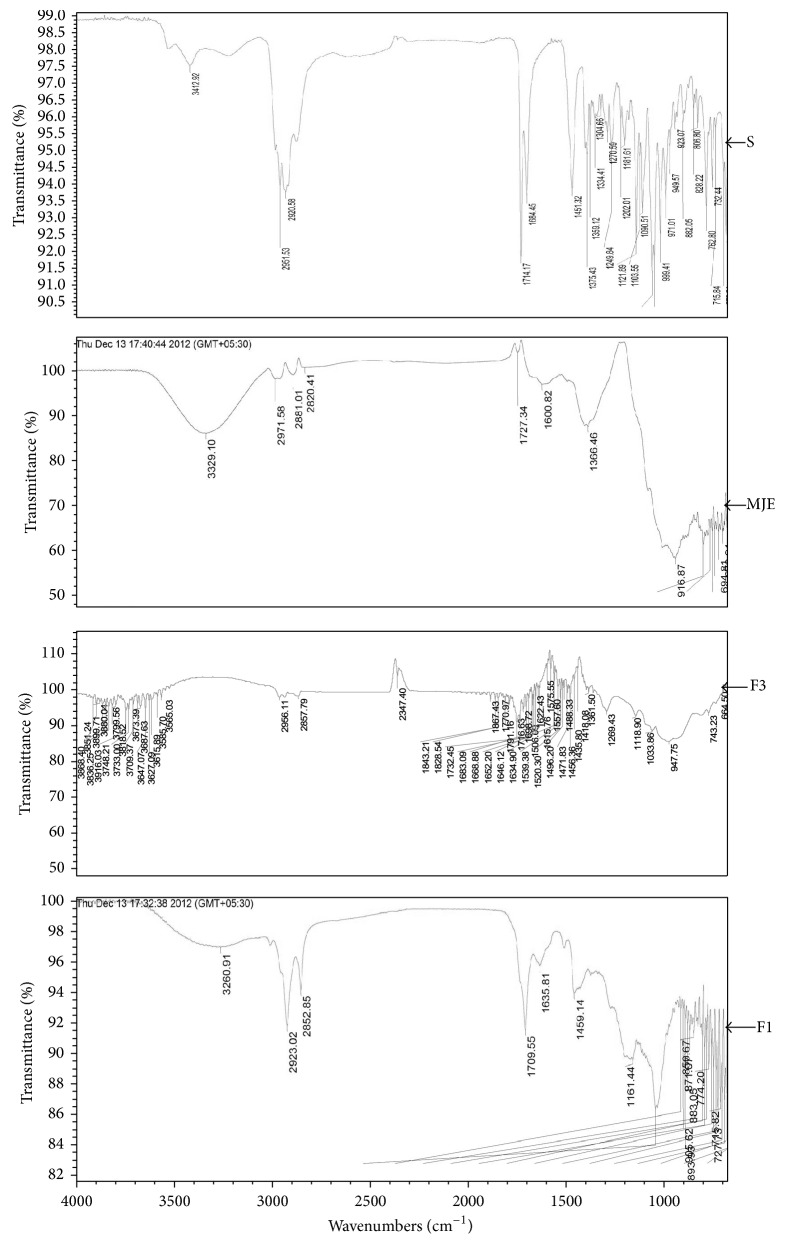
IR spectra of standard, MJE, and F1 and F3 fractions.

With the help of ATR (FTIR) analysis, bands of the major functional groups in the compound can be observed. The transmission bands of standard, MJE, F1, and F3 have been tabulated in Table 1. F1 and standard IR spectra showed similar spectra (Figure 1). MJE and F3 showed similar peaks at 956.87 and 947.75, respectively (Figure 1), whereas this peak was absent in F1 fraction.

### 3.2. Wound Healing Activity

#### 3.2.1. Rate of Wound Contraction and Biochemical Analysis

**Figure 2:**
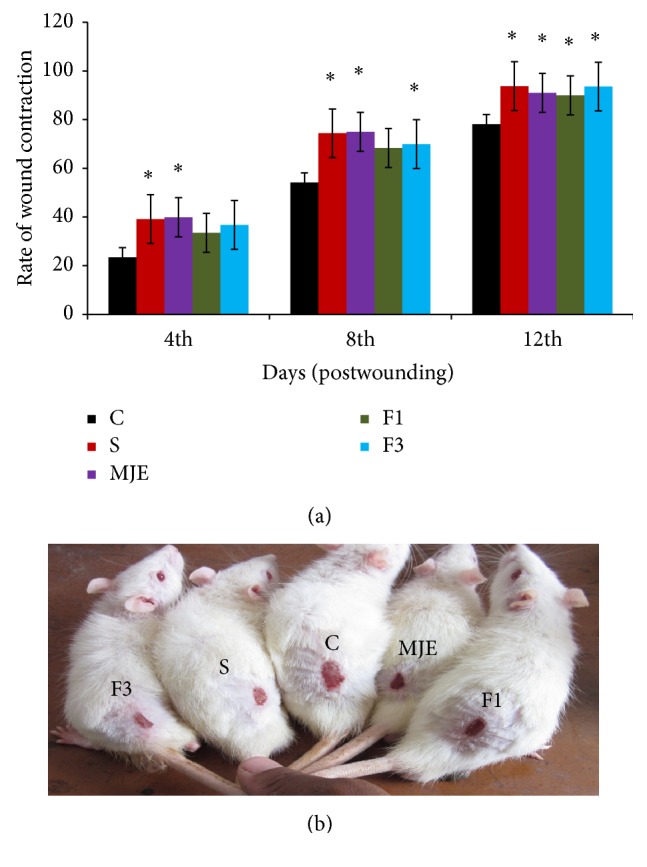
Rate of wound contraction of control(C), standard(S),* M. jalapa* crude extract (MJE), fraction 1 (F1) and fraction 3 (F3). (a) Rate of Wound contraction on 4th, 8^th^, and 12th day postwounding. (b) Pictorial representation of wound sizes on 12th day postwounding. Values are expressed as mean ± SD; *n* = 5; ^*^ *P* < 0.05 versus control and one-way ANOVA analysis followed by Dunnett's multiple comparison test was performed.

In the present investigation, a significant increase in wound contraction rate was observed in standard and MJE treated group as compared to control; however, no significant change was observed in F1 and F3 treated groups on the 4th postwounding day (Figure 2(a)). On 8th postwounding day, F3 treated group showed steady and gradual increase in wound contraction rate as compared to control; however, there was no significant change in F1 treated group (Figure 2(a)). On 12th postwounding day, the wound contraction rate of all the treated groups, that is, standard, MJE, F1, and F3, was significantly higher when compared to control group (Figures 2(a) and 2(b)).

In the present investigation, biochemical parameters showed different pattern of levels in fractions F1 and F3. SOD and CAT levels increased significantly (*P* < 0.05) in standard, MJE, and F1 as compared to control, whereas, in F3 treated group, there was no significant increase. CAT level increased significantly in standard and MJE treated groups as compared to control. F3 showed significant decrease (*P* < 0.05) in CAT level, whereas no change was observed in the level of SOD and POX when compared to control. POX levels decreased significantly in standard reference, MJE, and F1 when compared to control. Significantly, a low level (*P* < 0.05) of MDA was observed in standard, MJE, F1, and F3 as compared to control (Table 2). Hydroxyproline content was observed to be significantly increased (*P* < 0.05) in standard, MJE, and F3 as compared to control; however, there was no significant change in F1 treatment (Table 2). No significant change was observed in ascorbic acid content in standard, MJE, and F3 as compared to control. Ascorbic acid content was significantly decreased in F1 treatment as compared to control which supports the lower hydroxyproline content (Table 2).

A significant increase (*P* < 0.05) in COL 3A was observed in MJE, F1, and F3 as compared to control but reference drug showed no significant change (Figure 4). Upregulated expression of FGF 2 was observed in F1 when compared with that of control; however, no significant change was observed in the rest of the treatments. Significant upregulated expression of VEGF C was observed in all treated groups as compared to control.

In histological evaluation, uniform scab and ulcus layer were observed in standard, MJE, F1, and F3 on 8th postwounding granulation tissue (Figure 5, (b)A–(e)A). No uniformity was observed in control on 8th day granulation tissue (Figure 5, (a)A). In histological sections of 8th postwounding day (40x), distinct blood capillaries are formed in MJE and F1, but standard and F3 showed more macrophage infiltration (Figure 5, (b)B–(e)B). More infiltration of inflammatory cells were observed in 8th post wounding day tissue section of F3 (Figure 5, (a)B). On 12th day granulation tissue histological sections, standard and F3 showed thin epithelial line formation with remodelling of collagen fibres and hair follicle-like blood capillaries regeneration (Figure 5, (b)C; (b)D; (e)C; and (e)D). No epithelial line formation was observed but hair follicle-like blood capillaries were visible in MJE on 12th postwounding day tissue (Figure 5, (c)C and (c)D). Neither regeneration of hair follicle-like blood capillaries nor epithelial formation was observed in F1; however, it showed thicker ulcus layer and more fibroblast (myofibroblast) infiltration as compared to standard, MJE, F3, and control on 12th postwounding day tissue section (Figure 5, (d)C and (d)D).

## 4. Discussion

Earlier study revealed that hydromethanolic extract of* M. jalapa* tuberous roots showed potential in enhancing the wound healing process [6]. Also, terpenoid and flavonoid fractions of tuberous root of* M. jalapa* showed increased antioxidant and antimicrobial activities than the crude extract of* M. jalapa* [8]. Thus, it became important to evaluate the effects of MJE and its fractions in wound healing process on excision model.

F1 of MJE showed presence of terpenoids and steroids as its major components. Presence of these components in* M. jalapa* has been reported [19, 20]. Chemical test of the spots of TLC chromatogram showed the presence of steroids and IR spectra of F1 and standard showed similar bending and stretching patterns. Alkyl group stretching and shouldering peak with this band was observed at 2923.02 cm^−1^ and 2852.85 cm^−1^ in F1 and in case of standard it was at 2951.53 cm^−1^ and 2920.58 cm^−1^. It also showed band at 1709.53 cm^−1^ in F1 and 1714.17 cm^−1^ in standard which indicates presence of 3-4 membered ring structure (Figure 1). Standard used was a triterpenoid having 4 membered ring structure. Thus, the IR spectra of F1 show the presence of triterpenoid nucleus. One of the spots corresponds to *β*-sitosterol. *β*-Sitosterol from tuberous root of* M. jalapa* was reported in the literature [19]. Flavonoids were the major components of F3 of MJE. Quercetin O-rhamnoside and rutin which were reported in tuberous root of* M. jalapa* [19] were not detected on TLC chromatogram in this study.

**Figure 3:**
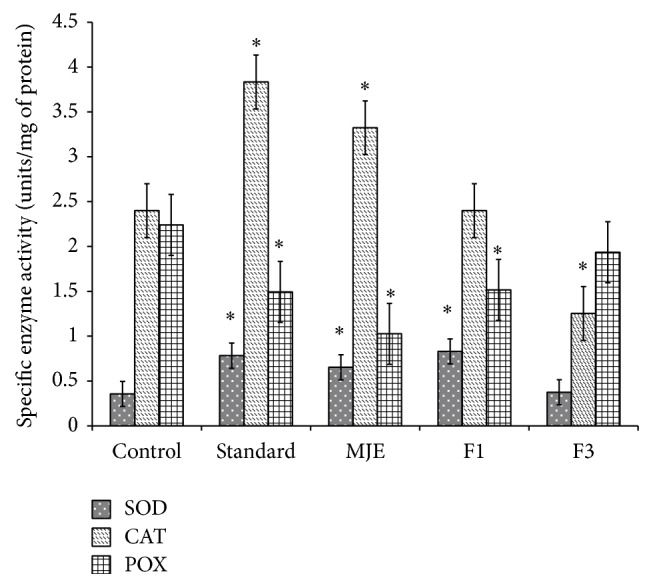
Effects of standard, MJE, and fractions on antioxidant enzymes on excision wound model. Control, standard, MJE = hydromethanolic extract of* M. jalapa*, F1 = fraction 1, and F3 = fraction 3. *n* = 5; values are expressed as mean ± SD; ^*^ *P* < 0.05 versus control and one-way ANOVA analysis followed by Dunnett's multiple comparison test was performed.

**Figure 4:**
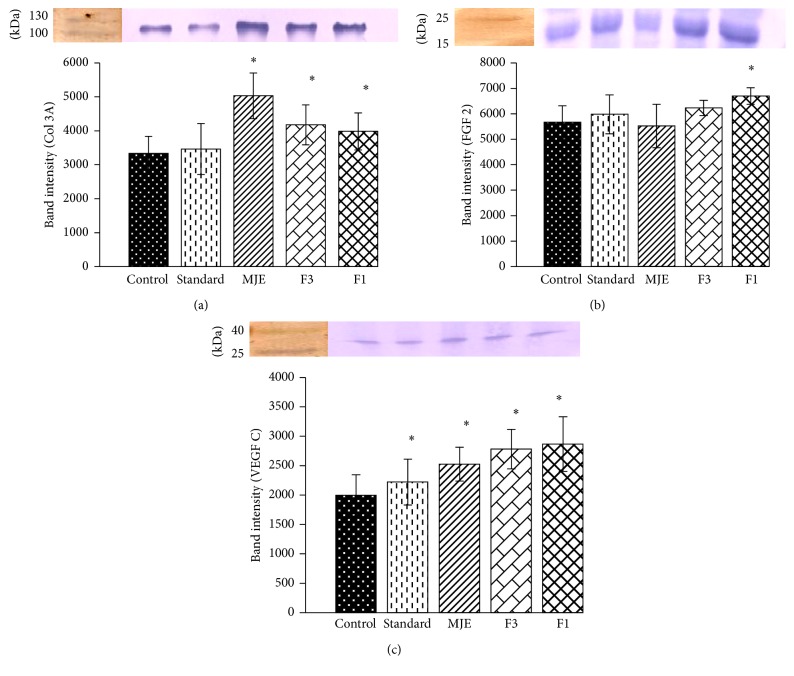
Effects of standard, MJE, and fractions on expression of growth factors (COL 3A, FGF 2, and VEGF C) on excision wound model. Control, standard, MJE = hydromethanolic extract of* M. jalapa*, F1 = fraction 1, and F3 = fraction 3. *n* = 5; values are expressed as mean ± SD; ^*^ *P* < 0.05 versus control and one-way ANOVA analysis followed by Dunnett's multiple comparison test was performed.

**Figure 5:**
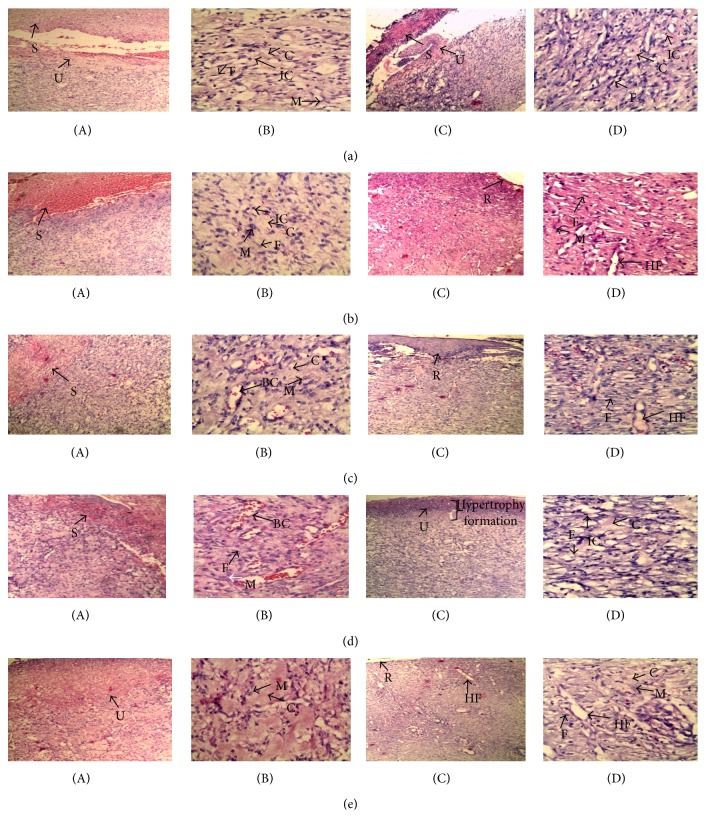
Histological sections of 8th (A-B) and 12th (C-D) postwounding day granulation tissue of excision wound. Hair follicle like blood capillaries formation (HF) and its movement to epithelial line was observed in 12th post wounding day tissue of standard (b)D, MJE (c)D and F3 (e)D as compared to control (a)D. Blood vessel (BC) formation was prominent on 8th postwounding day tissue of F1 (d)B as compared to control (a)B. Sections are control = (a)A–(a)D, standard = (b)A–(b)D, MJE = (c)A–(c)D, F1= (d)A–(d)D, and F3 = (e)A–(e)D. Macrophage (M), fibroblast (F), collagen (C), reepithelialisation (R), and inflammatory cells (IC) are shown in arrows in [10x (A and C)] and [40x (B and D)] HE stained sections.

Oxidative stress has been associated with more number of neutrophils in inflammatory phase (lasts for 2 days of after wounding, and, in some cases, it lasts for 2 weeks after) of wound healing; thus, to combat stress, increased antioxidant enzymes levels have been reported [21, 22]. Postwounding 3rd day to 12th day has been reported to be proliferative phase of healing; oxidative stress reduces with decline in neutrophils numbers. Fibroblast activation and collagen formation have been reported to be major event of the proliferative phase [23]. In the present comparative study, both enzymatic and nonenzymatic antioxidant systems of granulation tissue showed different pattern in both fractions. F3 showed lower antioxidant (SOD and CAT) level (Figure 3) as compared to F1 indicating that antioxidant enzyme levels normalise in F3 treated group. Also, reepithelialisation and wound contraction rate of F3 were observed to be faster as compared to F1 (Figure 5). These observations suggest that lowering of antioxidant levels by 8th day in F3 activates the fibroblast so as to increase the wound contraction rate and epithelialisation (Figures 2(b), 3, and 5). MDA and POX levels were high in standard, MJE, and F3 as compared to F1 (Figure 3 and Table 2), but F1 showed lower level of ascorbic acid as compared to control (Table 2). According to earlier report, ascorbic acid is considered to be important biochemical which induces lipid peroxidation and reactive aldehydes, helping in the stimulation of collagen gene expression in human fibroblasts [24]. Also, ascorbic acid is found to affect the activity of prolyl hydroxylases, rough endoplasmic reticulum enzymes that hydroxylate proline residues in nascent procollagen chains and allow folding of the chains into stable triple helix [25]. Thus, these observations suggest that increased levels of ascorbic acid and POX help in proper synthesis of collagen which is evident in the histological section of standard, MJE, and F3 (Figure 5). Also, increased expression of Col 3A supports the proper healing in standard and F3 fraction (Figure 4(a)).

F3 fraction was mainly found to be flavonoids, where flavone may be major component. Flavone has been considered to be antimicrobial agent synthesised by plant for its defence against microbial infection [26]. In wounds, microbial attack is very common process but histological observation showed low infiltration of inflammatory cells in the granulation tissues which indicates protective effect of F3 on wounds.

FGF 2 and VEGF growth factors are well documented to be involved in angiogenesis [27, 28]. Angiogenesis, the growth of new blood vessels, plays a key role in many physiological and pathological processes, such as ovulation, embryogenesis, wound repair, inflammation, malignant tumour growth, retinopathies, rheumatoid arthritis, and angiogenesis-dependent diseases. Both FGF 2 and VEGF C were upregulated in the fractions treatment (Figure 4). In the present study, increased expression of these growth factors was observed in F1 fraction and 8th day postwounding histological section also supported this change in F1 which may indicate prolonged proliferation phase in healing process. Further, on 12th day after wounding, histological section showed fibrous granulation tissue formation (more collagen deposition and no hair follicle-like blood capillaries formation) which may lead to hypertrophic scar formation in F1 treated group (Figure 5, (d)C and (d)D). Fibrous granulation tissue formation has been considered as pathological condition of wound healing process which indicates the chances of hypertrophy of skin [29].

Different parts of* M. jalapa* have medicinal uses in several regions of the world, such as anti-inflammatory [30], antiallergic [31] and as pain reliever [32]. F3 fraction with better antioxidant and antimicrobial activities [8] may have role in anti-inflammatory activity, which help in faster wound healing potential by lowering antioxidant enzyme level (reduce ROS formation which causes oxidative stress leading to delay in wound healing) and increasing ascorbic acid level for collagen remodelling and angiogenic activity. These changes have been observed in histological studies (Figure 5, (e)B and (e)D) which supports role of F3 fraction to enhance the wound healing process by formation of vascular granulation tissue (formation of hair follicle-like blood capillaries) and uniform layering of epithelial line which reduces the chance of scar formation.

## 5. Conclusion

Our results implicate that hydromethanolic crude extract of* M. jalapa* and flavonoid fraction enhance wound healing processes; however, terpenoid fraction may result in unregulated proliferation phase of wound healing and thereby it may lead to hypertrophic scar formation.

## Figures and Tables

**Table 1:** IR spectra transmission bands of standard, MJE, and fractions (F1 and F3).

Sample/standard	IR bands of the sample (cm^−1^)
Ursolic acid (standard)	3412.92, 2951.53, 2920.58, 1714.17, 1684.45, 1451.32, 1375.43, 1270.59, 1121.89, 1090.51

MJE	3329.10, 2971.58, 2881.01, 2820.41, 1727.34, 1600.02, 1366.46, 956.87

F1 fraction	3260.91, 2923.02, 2852.85, 1709.55, 1635.81, 1459.14, 1151.44

F3 fraction	2956.11, 1791.16, 1770.97, 1732.45, 1698.72, 1683.09, 1269.43, 947.75

**Table 2:** Effect of standard, MJE, and fractions on hydroxyproline, MDA, ascorbic acid content, and rate of wound contraction in excision wound model.

	Control	Standard	MJE	F1	F3
Hydroxyproline (mg/g of tissue)	5.88 ± 0.93	9.22 ± 1.41^*^	7.12 ± 0.87^*^	6.78 ± 0.38	7.307 ± 0.085^*^
Malondialdehyde (*µ*mol/g of tissue)	0.212 ± 0.074	0.1089 ± 0.094^*^	0.1066 ± 0.044^*^	0.0744 ± 0.054^*^	0.1106 ± 0.099^*^
Ascorbic acid (mg/g of tissue)	43.945 ± 4.71	58.375 ± 8.58	44.21 ± 1.12	32.14 ± 5.54^*^	51.11 ± 4.82

Standard: silver sulfadiazine, MJE: hydromethanolic extract of *M.  jalapa*, F1: fraction 1, and F3: fraction 3, *n* = 5; values are expressed as mean ± SD; ^*^ *P* < 0.05 versus control and one-way ANOVA analysis followed by Dunnett's multiple comparison test was performed.
